# MiR-216a reduces apoptosis of pulmonary microvascular endothelial cells in COPD by targeting DNMT1

**DOI:** 10.18332/tid/171357

**Published:** 2023-10-10

**Authors:** Ling Lin, Qing Song, Wei Cheng, Cong Liu, Aiyuan Zhou, Zijing Zhou, Ping Chen

**Affiliations:** 1 Department of Respiratory and Critical Care Medicine, the Second Xiangya Hospital, Central South University, Changsha, China; 2 Research Unit of Respiratory Disease, Central South University, Changsha, China; 3 Diagnosis and Treatment Center of Respiratory Disease, Central South University, Changsha, China; 4 Department of Respiratory and Critical Care Medicine, the Xiangya Hospital, Central South University, Changsha, China

**Keywords:** chronic obstructive pulmonary disease, MiR-216a, DNMT1, pulmonary microvascular endothelial cells, apoptosis

## Abstract

**INTRODUCTION:**

Abnormal apoptosis of pulmonary microvascular endothelial cells (PMVECs) participates in the pathogenesis of COPD. Studies have shown that microRNAs (miRNAs) contribute to the pathogenesis of pulmonary diseases by regulating cell apoptosis. The present study aimed to investigate the effects of miR-216a in cigarette smoke extract (CSE)-induced apoptosis of PMVECs in COPD and explore the potential mechanisms.

**METHODS:**

The emphysema model mice were treated with CSE and CS exposure. The expression of miR-216a and DNA methyltransferase 1 (DNMT1) was assessed in emphysema mice and COPD patients. The miR-216a mimic and Lenti-DNMT1 were transfected into PMVECs to identify the underlying mechanisms. The expression levels of miR-216a and DNMT1 were detected by real-time quantitative polymerase chain reaction (RT-qPCR) or Western blot. Moreover, cell apoptosis was examined by flow cytometry assays.

**RESULTS:**

The results show that the expression of miR-216a was decreased, whereas the expression of DNMT1 was increased in the lung tissue of emphysema mice and COPD patients. In addition, the expression of miR-216a was significantly reduced in CSE-treated PMVECs, and the overexpression of miR-216a attenuated CSE-induced PMVEC apoptosis. Furthermore, the expression of DNMT1 was increased in the CSE-induced PMVECs and then was reduced after the overexpression of miR-216a in the CSE-stimulated PMVECs. Luciferase reporter assays confirmed the target reaction between miR-216a and DNMT1. Also, the overexpression of DNMT1 was able to reverse the anti-apoptotic effect of miR-216a in CSE-induced PMVECs.

**CONCLUSIONS:**

The results indicate that miR-216a may play a crucial role in CSE-induced apoptosis by directly regulating its target gene DNMT1 in COPD. It provides insights into the function of MiR-216a/DNMT1 as a potential molecule in COPD.

## INTRODUCTION

Chronic obstructive pulmonary disease (COPD) is a common chronic respiratory disease characterized by recurrent respiratory symptoms and irreversible airflow limitations^1^. With the progress of the disease, the quality of life of the patients decreases dramatically, which causes a heavy economic burden for patients and is by far the most serious chronic respiratory disease^2^. However, the exact pathogenesis and treatment of COPD remains unclear.

Smoking is a major risk factor for the development of COPD. Cigarette smoke contains free radicals and oxidative compounds, which could injure lung tissue by inducing apoptosis and oxidative stress^3,4^. Studies have found that the apoptosis of pulmonary vascular endothelial cells was significantly increased in COPD patients and COPD mice. It has been shown that the inhibition of pulmonary vascular endothelial cell apoptosis can slow down or partially reverse the severity of emphysema in COPD animal models^5-7^. These results suggest that abnormal apoptosis of pulmonary vascular endothelial cells was involved in the development of COPD. However, the mechanism of apoptosis in pulmonary microvascular endothelial cells (PMVECs) needs to be further studied.

MicroRNAs (miRNAs) are a class of endogenous non-coding RNAs with regulatory functions found in eukaryotes which modulate gene expression by binding to complementary sequences in the coding or the 3'-untranslated region (UTR) of target mRNAs^8^. Multiple studies have shown that microRNAs participate in oxidative stress, inflammation, apoptosis and other physiological activities^9,10^. It also indicated that the expression of miRNAs may change in lung tissue of human and mice exposed to cigarette smoke (CS)^11,12^. The preliminary part of our study involved the use of microarray analysis on lung tissues of CS and cigarette smoke extract (CSE)-induced emphysema mice and control mice, we discovered that several miRNAs were differentially expressed in lung tissues, among which miR-216a was down-regulated in emphysema mice. Previous studies have demonstrated that miR-216a participates in the apoptosis of endothelial cells, for instance, Wang et al.^13^ indicated that it reduced miR-216a expression and enhanced the expression of cleaved-caspase-3 and other apoptosis-related proteins in human microvascular endothelial cells (HMVECS). In addition, the overexpression of miR-216a could reduce the apoptosis of human retinal microvascular endothelial cells^14^. Furthermore, current studies found that miR-216a plays a protective role in the development of lung diseases. For example, miR-216a could attenuate lipopolysaccharide-induced acute lung injury^15^ and protect human bronchial epithelium (HBE) cells from hydrogen peroxide (H2O2)-induced oxidative stress in asthma^16^. However, the mechanism by which miR-216a regulates the apoptosis of pulmonary endothelial cells in COPD remains unclear.

DNA methyltransferase 1 (DNMT1) is one of the DNA methyltransferases in mammalian cells, which can regulate the DNA methylation and be involved in cell cycle regulation, DNA repair, cell apoptosis, etc.^17,18^. In addition, DNMT1 is associated with the occurrence and development of lung diseases. The expression of DNMT1 in the lung tissue of COPD patients increased and DNMT1 can mediate the hypermethylation of the Bcl-2 promoter, thus enhancing apoptosis in the lung tissue of CSE induced emphysema mice^19^. Using the online database Targetscan 7.1 to predict downstream target genes of miR-216a, the results presented that DNMT1 is one of the target genes of miR-216a.

Hence, we proposed the hypothesis that miR-216a may reduce apoptosis in CSE-induced PMVECs in COPD. The purpose of this research was to explore whether miR-216a could alleviate the apoptosis of pulmonary microvascular endothelial cells in COPD by targeting DNA methyltransferase 1 (DNMT1).

## METHODS

### Study participants

The lung tissue was obtained from the control participants (N=7) and COPD patients (N=4) undergoing lung resection for lung cancer. All participants were studied at the Cardiothoracic Surgery Department at the Second Xiangya Hospital (Hunan, China). COPD was diagnosed when a ratio of forced expiratory volume in 1 second to forced vital capacity (FEV1/FVC) <0.70 after inhaling a bronchodilator. The study excluded patients with asthma, bronchiectasis, pulmonary fibrosis, pneumonia and other severe heart and lung diseases.

### Preparation of CSE

CSE was prepared as described previously20. Firstly, five cigarettes and half a cigarette (Furong, Changde Cigarette Company, Hunan, China) were burned for animal and cell experiments separately, and the smoke was bubbled through 10 mL PBS using a vacuum pump and a modified syringe-derived apparatus to obtain 100% CSE. Secondly, the CSE solution was filtered through a 0.22 μm pore-size filter. Finally, the 100% CSE solution was diluted with PBS to the required concentration for the animal and cell experiments.

### Animals’ model

Sixteen C57BL/6J male mice (20–22 g, 6 weeks) were purchased from Hunan Slyke Jingda Laboratory Animal corporation (Hunan, China). Mice were randomly divided into two groups (n=8 for each group): the control group, and emphysema mice (CS-and CSE-induced) group. The mice in the control group were maintained in fresh air and given an intraperitoneal injection of 0.01 mL/g phosphate-buffered saline (PBS) on days 0, 11 and 22. The methods were to establish emphysema mice, as described previously^18^. The emphysema mice model group were exposed to cigarette smoke for 2 cycles per day (1 hour per cycle), six cigarettes per cycle, 5 days per week for 4 consecutive weeks in a sealed box with ventilation holes and were intraperitoneally injected with 0.01 mL/g 100% CSE on days 0, 11 and 22. Cigarette exposure and CSE treatment were not implemented on the same day. On day 28, mice were killed and lung tissue was dissected and divided into two parts. The right lung tissue was placed in a liquid nitrogen tank for subsequent use for protein and RNA extraction while the left lung tissue was fixed with formalin.

All mice were fed and kept in a clean unit at 23 ± 2°C, 50 ± 10% relative humidity and 12 h rhythm of light and dark in the experimental animal center of the Hunan provincial people’s hospital (Hunan, China).

### Lung tissue morphometry and immunohistochemistry

The left lung tissue of the mice, after paraformaldehyde fixation, was embedded in paraffin wax and then cut into 3.5 µm thick sections which were stained with hematoxylin and eosin (HE). The mean linear intercept (MLI), destructive index (DI) and mean alveolar septal thickness (MAST) were measured to evaluate the morphological changes in the lung tissues. MAST was assessed by averaging 400 measurements in sections by microscopy at 400 × magnification. MLI represents the interalveolar septal wall distance and is the most widely accepted method to assess the extent and severity of emphysema. It is measured by dividing the length of a line through the lung part by the total number of intercepts counted within that line (×100 magnification). DI is a reliable index for determining alveolar destruction, indicating the percentage of alveolar destruction in lung tissue. It is measured by dividing the number of destroyed alveoli by the total number of alveoli counted^19,20^. Five representative and non-overlapping regions were selected from each lung section for measurement. The lung sections were also used for immunohistochemistry. 0.3% H2O2 was used to fix lung sections for 10 min after antigen retrieval in citrate buffer for 10 min in a microwave. Then, lung sections were incubated with anti-DNMT1 (ab188453, Abcam, Britain, 1:1000) at 4°C overnight. Next, lung sections were incubated with goat anti-rabbit IgG antibodies conjugated to peroxidase for 30 min at room temperature. Finally, diaminobenzidine was added and hematoxylin was applied for counterstaining. The protein expression levels were classified into the following grades according to the staining intensity: 1) no staining, 2) weak staining, 3) moderate staining; and 4) strong staining. The staining areas were scored as 0 (0%), 1 (1–10%), 2 (11–50%), or 3 (51–100%). The staining index was measured as the product of the proportion of positive cells and the staining intensity score^18^. Five different representative non-overlapping fields were selected for each lung section and an average score was calculated.

### TUNEL analysis and Immunofluorescence for CD31

The lung tissue apoptosis of mice was texted using the terminal deoxynucleotidyl transferase-mediated dUTP nick end labelling (TUNEL) detection kit (G1501, Servicebio, China) according to the manufacturer’s instructions. The lung tissue of the mice was fixed with 4% paraformaldehyde overnight at room temperature and embedded in paraffin. Then sections were incubated with CD31 (Servicebio, GB11063-2, China, 1:200) or SPC (Servicebio, GB114059, China, 1:200) at 4°C overnight. Lung sections were incubated with HRP-labelled goat anti-mouse IgG antibody (Servicebio, GB25301, China, 1:400) or HRP-labelled goat anti-rabbit IgG antibody (Servicebio, GB21303, China, 1:300) for 60 min at room temperature. Then, reaction solution and 4’,6-diamidino-2’-phenylindole (DAPI) (G1012, Servicebio, China) were used to counterstain the nuclei. Finally, the sections were observed under a fluorescence microscope (Nikon Eclipse C1, Japan) after being sealed.

### Arraystar miRNA array

Trizol reagent (Life Technologies Corporation, CA, USA) was used to lyse and isolate mouse lung tissue RNA and determine RNA quality. The RNA labelling reaction (Quick Amp Labeling Kit, One-Color, p/n 5190–0442, Agilent Technologies, CA, USA) and purification (RNeasy Mini Kit, p/n 74,104, Qiagen, Hilden, Germany) were used and solid-phase oligonucleotides with the same sequence as the target miRNA were applied to determine whether they were fluorescent- or biotin-labelled cDNA. Then, labelled/amplified cRNA was hybridized using the Agilent Gene Expression Hybridization Kit (p/n 5188–5242, Agilent). The fluorescence intensity of each well was measured for the subsequent analysis of miRNA expression levels in each well. Finally, the corresponding instruments were used to scan and analyze chip data.

### Isolation, culture, and identification of PMVECs

The mice PMVECs were isolated by magnetic-activated cell sorting. Briefly, the mice lung tissue was isolated under a sterile condition and marginal lung tissue was cut within 3 mm into small pieces. Then, the lung tissue was digested with type I collagenase solution (American Sigma) at 37°C for 45 min, before being centrifuged and filtered using 100 µm and 40 µm cell mesh. Next, CD31+ magnetic beads (Mildteny, Germany) were used and solutions were incubated at 4°C for 15 min. Finally, the cells were passed through an LS sorting column (Mildtenny, Germany) and centrifuged to obtain PMVECs. PMVECs are grown in 5% CO2 in endothelial cell culture medium at 37°C (Sciencell, USA). Higher purity endothelial cells were obtained after seven days.

After that, the PMVECs were identified by immunofluorescence staining with von Willebrand factor (vWF). Briefly, PMVECs were seeded in 24-well plates and blocked with 10% bovine serum albumin in 0.5% TritonX-100/PBS, before being incubated with the primary antibody of vWF (SA00006-4, Proteintech, USA) at 4°C for 12 h. Then, the cells were incubated with second antibody at room temperature for 2 h and stained with DAPI (G1012, Servicebio, China). Finally, fluoromount-G (0100-01, SourthernBiotech, USA) was added to the glass and a fluorescence microscope was used for analysis (OLYMPUS, Japan).

### Cell transfection

The PMVECs were seeded into 6-well plates until they reached 70–80% confluence. According to the manufacturer’s instructions, we used lipofectamine 3000 (Invitrogen, CA, USA) transfected miR-216a mimic, NC mimic (RiboBio Corporation, Guangzhou, China) into PMVECs for 24 h and then the PMVECs were treated with 2.5% CSE for 24 h. The PMVECs were seeded into a six-well plate and when the density reached 30–40%, an MOI value of 100 was used, virus infection enhancement solution, overexpression of DNA methyltransferase 1 (DNMT1) (lentivirus-DNMT1) and lentiviral vector (Genechem Corporation, Shanghai, China) were added; the solution was changed after 16 h of infection and the viral infection was observed under a fluorescence microscope after 72 h. Solutions were used for subsequent experiments.

### Western blot analysis

Total proteins were extracted from lung tissue and cells using RIPA lysis buffer (Beyotime, Shanghai, China). The protein concentrations of all were measured by a BCA protein assay kit (Thermo Fisher Scientific, Waltham, MA, USA). Equal amounts of proteins were separated using SDS–PAGE, transferred to polyvinylidene fluoride (PVDF) membranes and then incubated with the primary antibodies for Bax (14796, CST, USA, 1:1000), Bcl-2 (#3498, CST, USA, 1:1000), Cleaved caspase-3 (#9661, CST, USA, 1:1000), GAPDH (10494-1-AP, Proteintech, USA, 1:5000), DNMT1 (ab188453, Abcam, Britain, 1:1000) and β-actin (AC038, ABclonal, China, 1:10000) at 4°C for 24 h. Then, the membranes were incubated with HRP-conjugated goat anti-rabbit IgG (SA00001-2, Proteintech, USA, 1:10000) or HRP-conjugated goat anti-mouse IgG (SA00001-1, Proteintech, USA, 1:10000) at room temperature for 1 h. Finally, the protein bands were detected by an enhanced chemiluminescence detection system (BIO-RAD, California, USA).

### Real-time quantitative PCR (RT-qPCR)

Total RNA was extracted from lung tissue of mice and cells by using Trizol reagent (Invitrogen, Carlsbad, CA, USA). RNA was reverse-transcribed using the RevertAid First Strand cDNA Synthesis Kit (K1622, Thermo, USA). Then, qPCR was performed with SYBR green master mix (11184ES08, Yeasen, China) following the manufacturer’s instructions. The reverse transcription of miR-216a used the stem-loop method. The primers for miR-216a and U6 (including mice and human) were designed and constructed by RiboBio corporation (Guangzhou, China). Each PCR analysis was performed in triplicate. U6 was used as an internal control.

### Flow cytometry analysis

Briefly, Annexin V and Propidium Iodide (PI) staining (Thermo, USA) was used to examine apoptosis in PMVECs. According to the manufacturer’s instructions, PMVECs were transferred into 200 µL binding buffer and incubated with Annexin V for 15 min. Finally, the cells were incubated with the PI for 10 min. In order to set gates and establish appropriate compensation settings, cells were incubated with Annexin V alone, PI alone or the blank. The apoptosis rate of PMVECs in this study was analyzed for Annexin V-positive and PI-negative cells.

### Luciferase assay

After predicting the targets of miR-30b from the online databases: TargetScan 7.1 (https://www.targetscan.org/cgi-bin/targetscan/mmu_71/targetscan.cgi?mirg=mmu-miR-216a-3p), DNMT1 is one of target genes of miR-216a. Wild-type and mutant mouse DNMT1 (DNMT1-WT and DNMT1-MT) plasmids (HonorGene company, Hunan, China) were constructed. Secondly, mmu-miR-216a mimic or NC mimic, DNMT1-WT and DNMT1-MT plasmids (1 µg/well in 6 well plates) were transfected into PMVECs in 24-well plate for 24 h by using lipofectamine 3000. Finally, firefly and Renilla luciferase activities were measured using the dual-luciferase assay kit (Keygen company, Jiangsu, China) following the instructions. The luciferase activity was expressed as a ratio of firefly luciferase to Renilla luciferase units.

### Statistical analysis

Statistical analysis was performed using a software package (SPSS 26.0, SPSS Inc., Chicago, IL, USA). The continuous variables, normally distributed and homogeneous variance, were performed using independent-sample t-test or analysis of variance. LSD-t test is used for pairwise comparison between groups. The non-parametric test was used for non-normal distribution or uneven variance. A p<0.05 was considered to be statistically significant.

## RESULTS

### The expression of miR-216a and DNMT1 in emphysema mice and COPD patients

*The expression of miR-216a and DNMT1 in the lung tissue of emphysema mice*

The lung tissue of emphysema mice which was exposed to CS and CSE showed enlarged alveolar spaces, thinner alveolar septal and destruction of alveolar walls compared to the control group (Supplementary file Figure 1A). In addition, the DI and MLI were enhanced in emphysema mice, whereas the MAST was reduced in emphysema mice. Also, the expression of cleaved caspase-3 was increased and anti-apoptotic protein Bcl2 was decreased in emphysema mice (Supplementary file Figure 1E). Moreover, immunofluorescence staining of lung tissue with TUNEL and the endothelial cell marker, CD31, revealed that the increased apoptosis was observed in the PMVECs of emphysema mice than in control mice (Supplementary file Figure 1B). MicroRNA array analysis was used to measure the differentially expressed microRNAs in the lung tissues of emphysema and control mice. In total, 1186 distinct microRNAs transcripts were detected in the lung tissues of all subjects. In emphysema mice, 12 microRNAs were differentially expressed (≥2-fold change and p<0.05) compared to the control group (Supplementary file Figure 1C). The expression of miR-216a in another two sets of lung tissue samples was validated using real-time PCR. The results presented that the expression of miR-216a was significantly downregulated in emphysema mice and it was consistent with the result of the microarray data (Supplementary file Figure 1D). Besides, the expression of DNMT1 was significantly increased in emphysema mice compared with the control group in immunohistochemistry and western blot (Supplementary file Figures 1 E–F).

*The expression of hsa-miR-216a and DNMT1 in lung tissue of COPD patients*

To validate the expression of hsa-miR-216a and DNMT1 in COPD patients, we collected the lung tissues of healthy control participants and COPD patients. The clinical characteristics of the participants are shown in Table 1. There was no significant difference in sex, age, or body mass index. Compared with the control group, the expression of miR-216a was significantly decreased in COPD patients (Supplementary file Figure 1G). Besides, the expression of DNMT1 was significantly increased in COPD patients compared with the control group using western blot and immunohistochemistry (Supplementary file Figures 1 H–I).

**Table 1:** Clinical characteristics of the patients, 2021 (N=11)

*Characteristics*	*Control participants (N=7) Mean ± SD*	*COPD patients (N=4) Mean ± SD*	*p*
**Age** (years)	57.3 ± 6.3	59.1 ± 7.7	0.686
**Sex,** n (%)			0.819
Male	4 (57.1)	2 (50.0)	
Female	3 (42.9)	2 (50.0)	
**BMI** (kg/m^2^)	22.6 ± 4.4	23.5 ± 4.5	0.526
**Smoking** (pack/years), median (IQR)	0 (30)	22.5 (56.2)	0.680
**Pulmonary function**			
FEV1	2.2 ± 0.5	1.5 ± 0.6	**0.038**
FEV1 %pred	80.4 ± 14.5	58.0 ± 10.0	**0.012**
FEV1/FVC	82.3 ± 12.1	56.2 ± 9.4	**0.006**

BMI: body mass index. COPD: chronic obstructive pulmonary disease. FEV1: forced expiratory volume in one second. FVC: forced vital capacity.

### CSE down-regulated the expression of miR-216a in PMVECs

**Figure 1:**
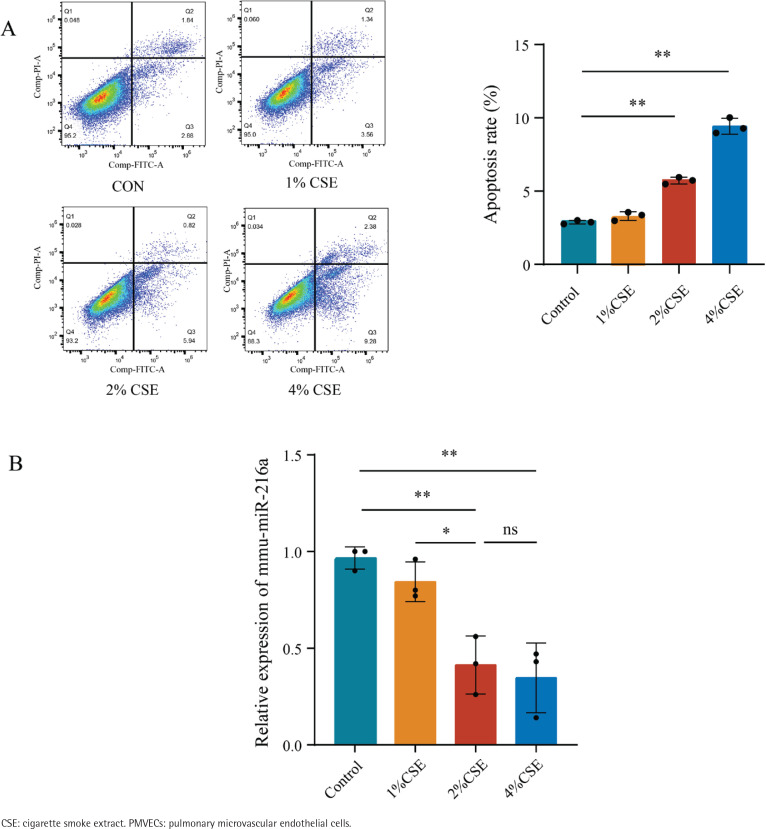
CSE downregulated the expression of miR-216a in PMVECs, 2022: A) The apoptosis of different concentrations (0%, 1%, 2% and 4%) of CSE-induced PMVECs was detected by flow cytometry; B) The expression of miR-216a in different concentration (0%, 1%, 2% and 4%) of CSE-induced PMVECs

After exposing PMVECs to different CSE concentrations (0, 1, 2 and 4%) separately for 24 h, the results of flow cytometry analysis revealed that the early apoptosis rate initially increased as CSE concentrations gradually increased from 2% to 4% (Figure 1A). It reflected a dose-dependent effect of CSE on apoptosis in PMVECs. However, the expression of miR-216a was reduced at CSE concentrations of 2% and 4% (Figure 1B). Based on the above, we decided to expose cells to 2% CSE for 24 h for the following experiments.

### MiR-216a attenuated apoptosis of CSE-induced PMVECs

**Figure 2:**
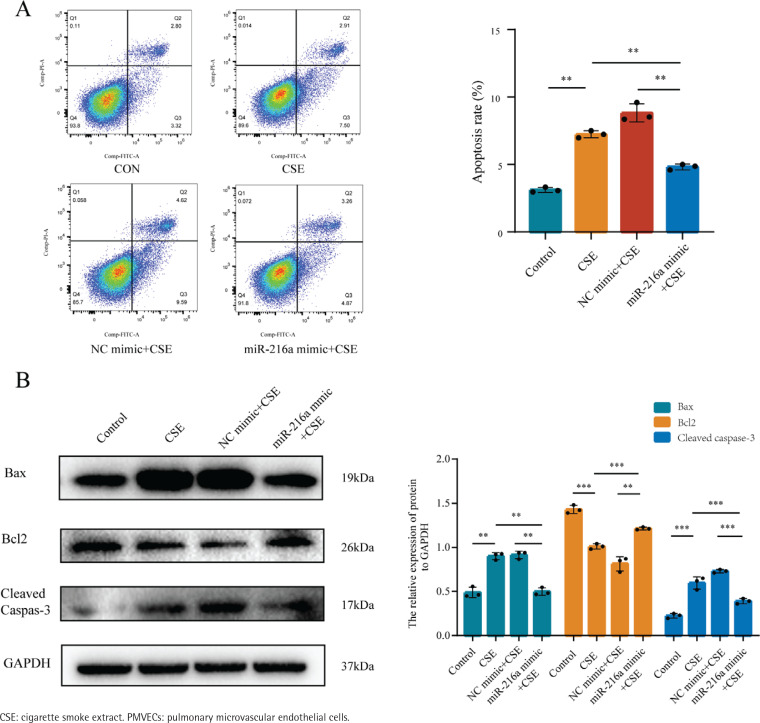
MiR-216a attenuated apoptosis of CSE-induced PMVECs, 2022: A) The apoptosis of CSE-induced PMVECs after transfected with miR-216a mimic detected by flow cytometry; CSE, 2% CSE; B) The apoptosis related protein expression in CSE-induced PMVECs after transfected with miR-216a mimic detected by Western blot; CSE, 2% CSE

To examine whether miR-216a plays a protective role on CSE-induced apoptosis of PMVECs, the cells were transfected with miR-216a mimic to achieve the overexpression of miR-216a in PMVECs (Supplementary file Figure 2). Furthermore, flow cytometry results showed significantly increased apoptosis rates in the CSE-treated PMVECs, whereas, the overexpression of miR-216a could significantly alleviate CSE-induced cell apoptosis (Figure 2A). In addition, the expression of Bax and Cleaved caspase-3 was decreased, while the expression of Bcl-2 in western blots was increased after PMVECs were treated with the overexpression of miR-216a (Figure 2B). These results implied that miR-216a was involved in regulating CSE-induced PMVECs apoptosis *in vitro*.

### DNMT1 is a target of miR-216a in PMVECs

**Figure 3:**
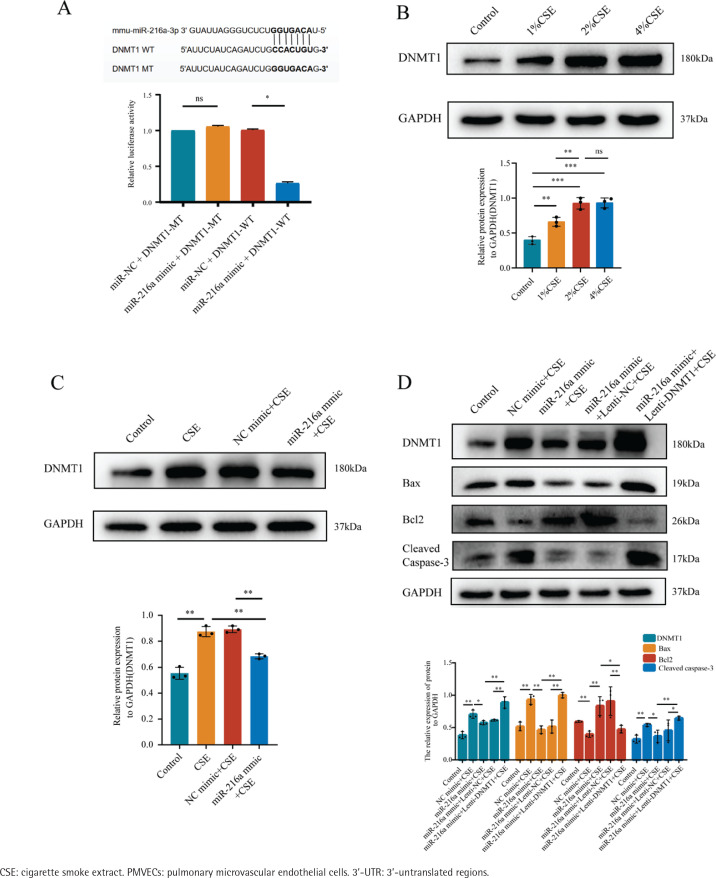
MiR-216a reduced apoptosis of CSE-induced PMVECs via targeting DNMT1, 2022: A) Luciferase reporter assay demonstrated that miR-216a could directly target 3’-UTR of DNM1; B) The expression of DNMT1 in different concentration (0%, 1%, 2% and 4%) of CSE-induced PMVECs detected by Western blot; C) The expression of DNMT1 in CSE-induced PMVECs after transfected with miR-216a mimic detected by Western blot; CSE, 2% CSE; D) The overexpression of DNMT1 could reverse the anti-apoptotic effect of miR-216a in CSE-induced PMVECs; the expression of DNMT1 and apoptosis-related proteins detected by Western blot; CSE, 2% CSE

To further identify the downstream molecules directly affected by miR-216a, we predicted the targets of miR-216a from the TargetScan database, which predicted that DNMT1 was a potential target gene of miR-216a. We constructed a luciferase reporter assay to verify that DNMT1 is the direct target of miR-216a. We co-transfected PMVECs with miR-216a mimic and scrambled miRNA (negative control) and the luciferase reporter vectors of DNMT1-WT and DNMT1-MT, which contained the wild type and mutant type potential binding sequences of miR-216a-3p in the 3’-UTR of DNMT1, respectively. It showed that luciferase activity in the DNMT1-WT and miR-216a mimic groups was significantly decreased. However, this effect was not observed in the DNMT1-MT + miR-216a mimic group (Figure 3A). Collectively, these results suggest that DNMT1 is regulated by miR-216a in PMVECs. In addition, the expression of DNMT1 gradually increased with the different CSE concentrations in PMVECs (Figure 3B). Enhancing the expression of miR-216a by transfecting with miR-216a mimic could downregulate the expression of DNMT1 in CSE-induced PMVECS (Figure 3C).

### The overexpression of DNMT1 reversed the anti-apoptosis effects of miR-216a in CSE-treated PMVECs

PMVECs were transfected with Lenti-NC and Lenti-DNMT1 for 72h. The results exhibited that the expression of DNMT1 was enhanced after PMVECs were transfected with Lenti-DNMT1. Also, the expression of Cleaved caspase-3 and Bax was increased, while the expression of Bcl-2 was decreased. The results reflected that the overexpression of DNMT1 could significantly increase the apoptosis of PMVECs (Supplementary file Figure 3). Finally, we co-transfected the miR-216a mimic and Lenti-DNMT1 into PMVECs. As expected, it decreased the expression of Bcl-2 and increased the expression of Bax and cleaved caspase-3 in the overexpression of DNMT1 and the miR-216a group compared with the overexpression of the miR-216a group in CSE-induced PMVECs. This indicated that the overexpression of DNMT1 could reverse the anti-apoptotic effects of miR-216a in CSE-induced PMVECs (Figure 3D).

## DISCUSSION

Studies have revealed the relation between cigarette smoke, cell apoptosis, and the development of emphysema^5,21-24^. Cigarette smoke can induce the apoptosis of macrophages, alveolar epithelial cell lines, vascular endothelial cells, etc.^25-27^. Consistent with this concept, the present study showed that CSE induced PMVECs apoptosis in a dose-dependent manner. In this study, the concentration of 2% CSE was selected as the standard intervention condition, which leading to a high apoptosis rate and moderate necrosis in the early stages. This is consistent with the principle of the Long et al.^28^ method of choosing CSE concentration for interventions in human pulmonary endothelia cells.

MiRNA dysregulation has been widely shown to be associated with the development of smoking-related lung disease. Several studies have reported the changes in miRNA expression in lung tissues obtained from COPD patients and animal models of COPD exposed to cigarette smoke^11,12,29^. Previous studies have demonstrated that miR-21^30^, miR-126^31^ and miR-34a^28^ play an important role in the pathogenesis and development of COPD. Our research group conducted microarray analysis of the lung tissues of CS and CSE exposure-induced emphysema mice and control mice and found that a few miRNAs were differentially expressed in the lung tissues of emphysema mice. Among these, miR-216a was down-regulated in emphysema mice and the expression of miR-216a was down-regulated in CSE-stimulated PMVECs. This reflected that miR-216a regulated the apoptosis of PMVECs and is in keeping with previous studies in which miR-216a was shown to be involved in cell apoptosis^32,33^. This study also indicated that the overexpression of miR-216a could attenuate the apoptosis of PMVECs, and down-regulated the expression of Bax and Cleaved caspase3, while it increased the expression of Bcl2. These results indicated that miR-216a played a protective role in the apoptosis of PMVECs. Although miR-216a has not been studied in COPD, miR-216a has been reported to play a protective role in other lung diseases, such as acute lung injury^15^ and asthma^16^. More importantly, a previous study demonstrated that miR-216a decreased and inhibited the viability of human microvascular endothelial cells and promoted the expression of Cleaved-caspase-3 and other apoptosis-related proteins in HMVECs of peripheral artery diseases^13^. In diabetic retinopathy, the overexpression of miR-216a reduced the apoptosis and improved the survival ability of human retinal microvascular endothelial cells under high glucose conditions^14^. However, it is crucial to discover its biological function by identifying the important mRNA targets of miR-216a and to facilitate further studies.

Using the prediction software TargetScan, DNMT1 was predicted to be the target gene of miR-216a. The luciferase report in this study demonstrated that miR-216a binds to the putative 3 ‘-UTR binding site of DNMT1. DNMT1, one of the major DNA methyltransferases in mammalian cells, is a large and highly dynamic enzyme that controls DNA methylation in cells. DNMT1 is involved in chromatin assembly, DNA repair, cell cycle regulation and apoptosis^17,18^. This study demonstrated that increased DNMT1 expression in the lung tissues of emphysema mice and COPD patients, as well as CSE-induced PMVECs, which is consistent with the results of Zeng et al.^19^, revealed that the expression of DNMT1 increased in lung tissues of COPD patients and emphysema mice. Our study aimed to verify the expression levels of DNMT1 in PMVECs and further investigated the role of DNMT1 in the apoptosis of CSE-stimulated PMVECs in COPD. Our study revealed that the overexpression of miR-216a reduced the expression of DNMT1 in PMVECs, resulting in a negative regulation between miR-216a and DNMT1. Following the overexpression of DNMT1 in PMVECs, the expression levels of Bax and Cleaved caspase-3 were enhanced, while the expression of Bcl2 was down-regulated. These results indicate that DNMT1 was involved in the apoptosis of PMVECs. This is in line with previous studies in a CS-exposed mouse model of emphysema in which oxidative stress was implicated in lung apoptosis via DNMT1-mediated hypermethylation of the Bcl2 promoter^19^. In addition, DNMT1 has been shown to participate in the regulation of endothelial apoptosis in other disease models. In the atherosclerosis model, the increased expression of DNMT1 in human umbilical vein endothelial cells (HUVECs) leads to the apoptosis of endothelial cells^34^. Besides, the up-regulation of DNMT1 in homocysteine-treated HUVECs aggravated endothelial cell apoptosis^35^. In addition, this study elucidated that the overexpression of DNMT1 could reverse the anti-apoptotic effect of miR-216a in CSE-induced PMVECs. As a result, it reflected that DNMT1 is involved in miR-216a induced apoptosis in PMVECs. As above, the study demonstrated the role of the miR-216a/DNMT1 axis in CSE-induced PMVECs apoptosis in COPD (Supplementary file Figure 4).

### Limitations

There are some limitations of our study. Firstly, our study elucidated the mechanism by which miR-216a reduced PMVECs apoptosis by regulating DNMT1 *in vitro*, therefore, more studies *in vivo* are needed in the future research. Secondly, although the specific mechanism by which DNMT1 regulates PMVECs apoptosis has not been explored, previous study^6^ demonstrated that DNMT1 could be involved in apoptosis by regulating hypermethylation of Bcl-2 promoter.

## CONCLUSIONS

Our results suggest that miR-216a was involved in CSE-induced PMVECs apoptosis in COPD. Furthermore, this effect may be associated with the regulation of its target, DNMT1. The miR-216a/DNMT1axis may be a crucial regulator of cellular apoptosis in COPD. Consequently, our study provides insights into the function of MiR-216a/DNMT1 as a potential molecule in COPD.

## Data Availability

The datasets supporting this research are included within the article.
