# Supplementary material for: MiR-216a reduces apoptosis of pulmonary microvascular endothelial cells in COPD by targeting DNMT1
Source: Tob Induc Dis. 2023 Oct 10;21:130. doi: 10.18332/tid/171357 (PMC10563178; doi:10.18332/tid/171357)
Supplement: Supplementary file 1 [file TID-21-130-s1.pdf]

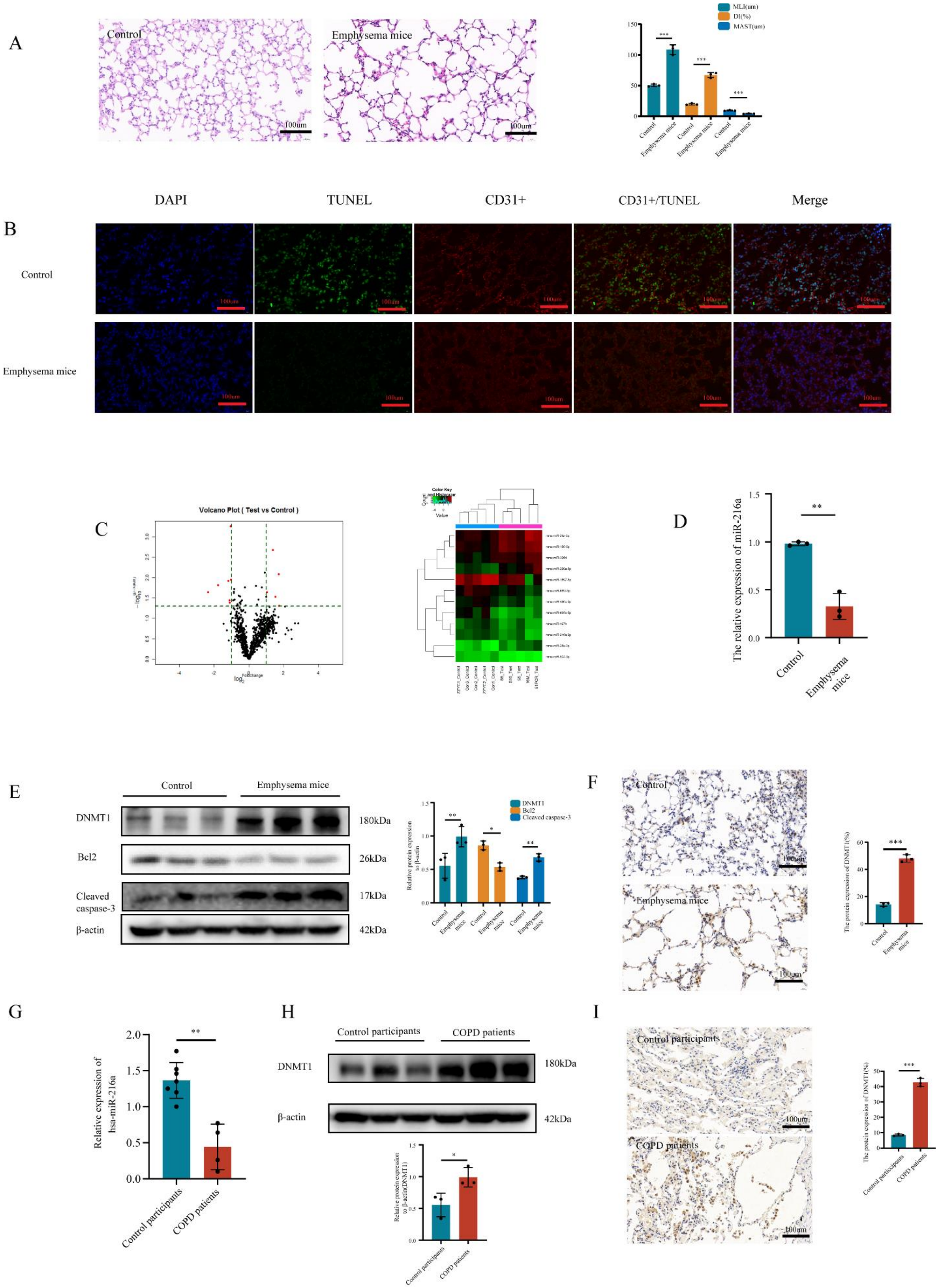

A
Control
Emphysema mice
100um
MLI(um)
DI(%)
MAST(um)
150
100
50
0
***
Control
Emphysema mice
B
DAPI
TUNEL
CD31+
CD31+/TUNEL
Merge
Control
Emphysema mice
100um
C
Volcano Plot (Test vs Control)
Color Key and Histogram
Value
D
The relative expression of miR-216a
1.5
1.0
0.5
0.0
**
Control
Emphysema mice
E
Control
Emphysema mice
DNMT1
Bcl2
Cleaved caspase-3
β-actin
180kDa
26kDa
17kDa
42kDa
Relative protein expression to β-actin
DNMT1
Bcl2
Cleaved caspase-3
**
*
F
Control
Emphysema mice
100um
The protein expression of DNMT1(%)
***
G
Relative expression of hsa-miR-216a
2.0
1.5
1.0
0.5
0.0
**
Control participants
COPD patients
H
Control participants
COPD patients
DNMT1
β-actin
180kDa
42kDa
Relative protein expression to β-actin(DNMT1)
*
I
Control participants
COPD patients
100um
The protein expression of DNMT1(%)
***

Supp. Figure 1: The expression of miR-216a and DNMT1 in lung tissue of emphysema mice and patient with COPD,2021. (A)HE staining for lung tissue in control mice and emphysema mice (n=3, per group) ; Scale bar, 100um(×400); emphysema mice was induced CS+CSE, control mice was treated with fresh air and PBS. (B) Representative immunofluorescent images of control mice and emphysema mice, lung sections stained for TUNEL (green), endothelial cell marker CD31 (red) and DAPI (blue). Scale bar, 100 µm (×600). emphysema mice was induced CS+CSE, control mice was treated with fresh air and PBS. (C) Hierarchical clustering and volcano plot of differential expressed miRNAs in emphysema mice compared to control mice (n=5, per group), emphysema mice was induced CS+CSE, control mice was treated with fresh air and PBS. (D) The verification of miR-216a expression in lung tissue of mice (n=3, per group), emphysema mice was induced CS+CSE, control mice was treated with fresh air and PBS. (E) Western blot detected the expression of DNMT1 in mice lung tissue (n=3, per group). (F) Immunohistochemistry detected the expression of DNMT1 in mice lung tissue (n=3, per group), Scale bar, 100um (×400); emphysema mice was induced CS+CSE, control mice was treated with fresh air and PBS. (G) The expression of miR-216a in control participants(n=7) and COPD patients(n=4). (H) The protein expression of DNMT1 in control participants(n=7) and COPD patients(n=4). (I) Immunohistochemistry detected the expression of DNMT1 in patient lung tissue;Scale bar, 100um(×400); Control participants (n=7) and COPD patients (n=4). COPD, Chronic obstructive pulmonary disease; DI, destructive index; MLI, mean linear intercept; TUNEL, Terminal deoxynucleotidyl transferase-mediated dUTP nick end labelling. *: $p<0.05$; ** : $p<0.01$.

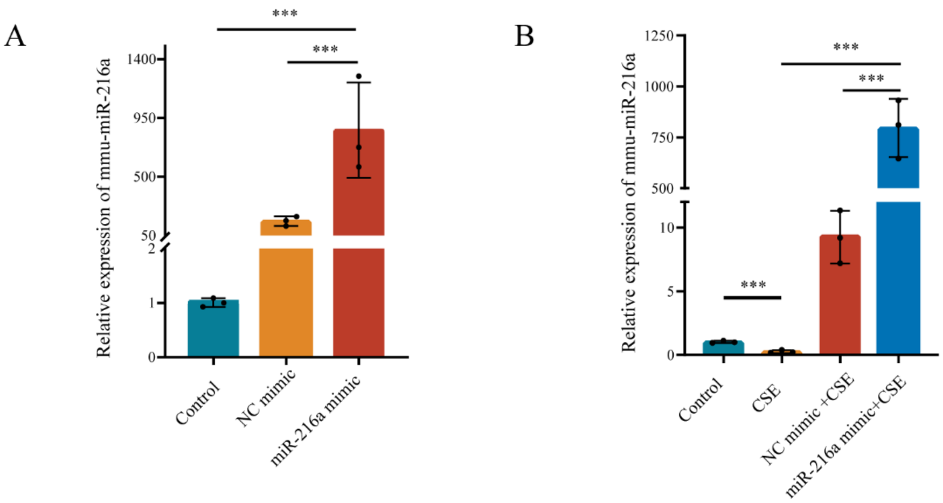

Supp. Figure 2: The expression of miR-216a after being transfected with miR-216a mimic,2022 (A)Verified transfection efficiency of miR-216a mimic in PMVECs after being transfected with miR-216a mimic; CSE, 2% CSE. (B)Verified the expression of miR-216a in CSE-induced PMVECs after being transfected with miR-216a mimic; CSE, 2% CSE. *** : $p<0.001$.

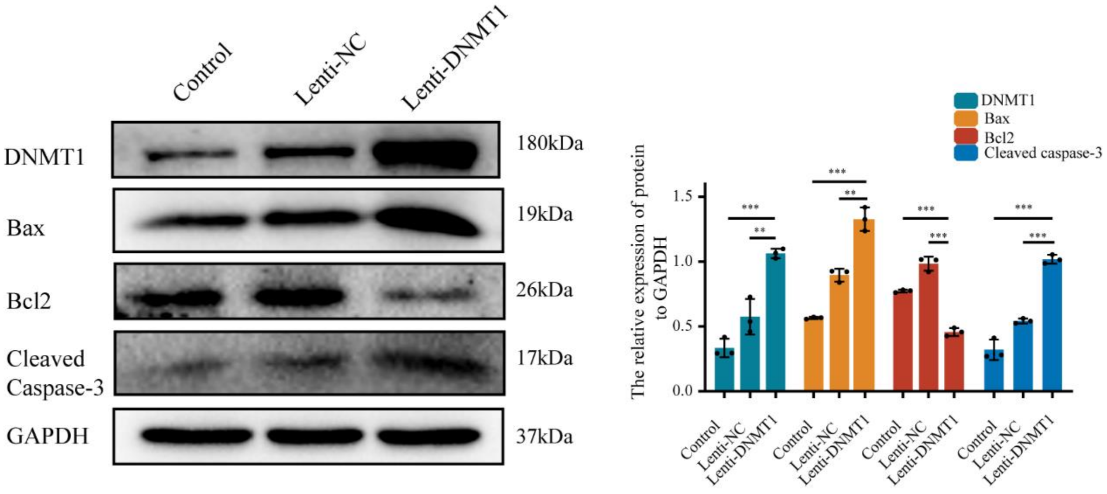

Supp. Figure 3: The overexpression of DNMT1 could increase the PMVECs apoptosis,2022 Abbreviations: Lenti-NC group: PMVECs was transfected with lentiviral vector; Lenti-DNMT1 group: PMVECs was transfected with lentiviral-DNMT1.The expression of DNMT1 was higher in Lenti-DNMT1 group than Control and Lenti-NC group in PMVECs, the expression of Bax and Cleaved Caspase-3 was higher but the expression of Bcl2 was reduced in Lenti-DNMT1 group than Control and Lenti-NC group in PMVECs. CSE, 2% CSE; ** : p＜0.01; *** : p＜0.001.

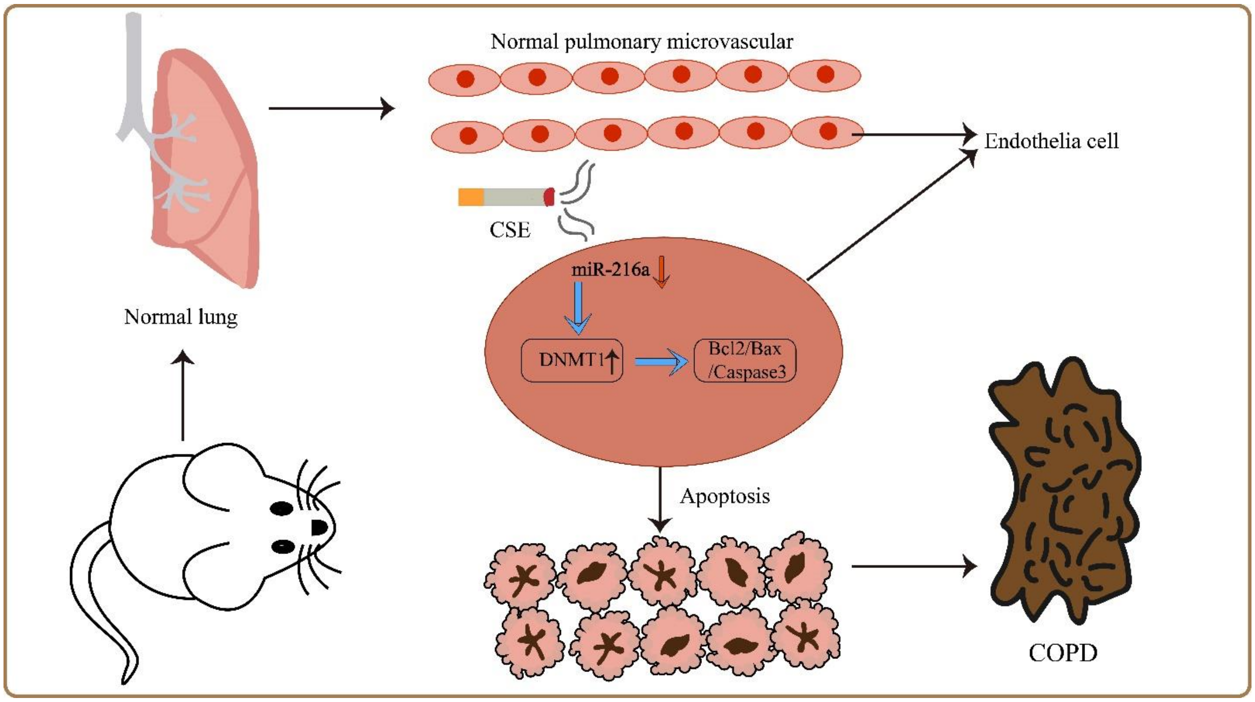

Supp. Figure 4: Flow chart and mechanisms of this study Abbreviations: COPD, Chronic obstructive pulmonary disease; CSE, Cigarette smoke extract.

© 2023 Lin L. et al.
